# Standardized therapies after ECMO program (STEP); a novel approach to pediatric post-ECMO care

**DOI:** 10.1051/ject/2024009

**Published:** 2024-09-20

**Authors:** Rebekah K.H. Shappley, Christen M. Holder, Constance E. Poplos, Pilar Anton-Martin, Thomas Spentzas, Toni M. Whitaker, Swati Karmarkar, Samir H. Shah, Hitesh S. Sandhu

**Affiliations:** 1 Department of Pediatrics, Division of Critical Care, University of Tennessee Health Science Center Memphis TN 38103 USA; 2 Le Bonheur Children’s Hospital Memphis TN 38103 USA; 3 Department of Pediatrics, Division of Neurology, University of Tennessee Health Science Center Memphis TN 38103 USA; 4 Neuroscience Institute, Le Bonheur Children’s Hospital Memphis TN 38103 USA; 5 University of Tennessee Health Science Center, College of Medicine Memphis TN 38103 USA; 6 Department of Pediatrics, Children’s Hospital of Philadelphia Philadelphia PA 19104 USA; 7 Department of Pediatrics, Division of Development Pediatrics, University of Tennessee Health Science Center Memphis TN 38103 USA; 8 Baylor College of Medicine Houston TX 77030 USA

**Keywords:** Pediatric ECMO, Follow-up care, Post-ECMO follow-up care, Intentional discharge pathway, ELSO, Neuro developmental follow-up

## Abstract

*Background*: The study objective was to characterize compliance with Standardized Therapy after ECMO Program (STEP), an intentional discharge pathway for extracorporeal membrane oxygenation (ECMO) survivors in a US pediatric hospital. *Methods*: The program identified pediatric ECMO survivors before discharge, appropriate consultations were reviewed and requested, families were educated on ECMO sequelae, and ECMO summaries were sent to pediatricians. Compliance with institutional post-ECMO guidelines was evaluated before and after STEP implementation. *Results*: We identified 77 ECMO survivors to hospital discharge (36 [46.8%] before and 41 [53.2%] after STEP implementation). There was a significant increase in complete (38.8% vs. 74.2%, *p* < 0.001) and time-appropriate neurodevelopmental testing (71.4% vs. 95.6%, *p* = 0.03). Significant increase in inpatient evaluations by neurology (52.7% vs. 75.6%, *p* = 0.03) and audiology (66.7% vs. 87.8%, *p* = 0.02), and in referrals for outpatient audiology (66.6 vs. 95.1%, *p* = 0.002), physical therapy (P.T.) (63.8% vs. 95.1%, *p* = 0.001), occupational therapy (O.T.) (63.8% vs. 95.1%, *p* = 0.001) and speech-language pathology (S.L.P.) (55.5% vs. 95.1%, *p* < 0.001) were noted. *Conclusion*: Implementing an intentional discharge pathway for pediatric ECMO survivors (STEP) successfully increases inpatient and outpatient compliance with hospital and Extracorporeal life support organization (ELSO) follow-up guidelines. It leads to timely and complete neurodevelopmental evaluation.

## Introduction

The expanding application of ECMO support has improved hospital discharge numbers and a growing realization that ECMO survivors have medical, developmental, and social needs that require early identification and structured follow-up [1–3]. ECMO has immediate risks (e.g., bleeding, thrombosis, cerebral hemorrhage, decreased hippocampus volume) and long-term deficits [4–9]. While many patients thrive, some may demonstrate sequelae of critical illness, such as post-intensive care syndrome, with or without sequelae of ECMO itself.

Children who survive ECMO are at risk for developmental delays and the “growing into deficits” phenomenon where effects of early subtle injuries are detected, especially when higher cognitive functions require those injured regions [2, 3, 10–13]. Neonates who develop appropriate motor skills may still be at risk for other deficits and need longer-term developmental follow-up. Other post-ECMO challenges include poor exercise tolerance, decreased respiratory function, and renal dysfunction [2]. Early identification and appropriate therapies can make a difference in long-term neurological outcomes, as shown by a randomized control trial that improved visuospatial memory in neonatal ECMO survivors after therapy [14].

Extracorporeal Life Support Organization (ELSO) first published follow-up guidelines in 1994 and updated them in 1997 and 2021 [1, 15]. A few studies examined the impact of a comprehensive follow-up approach on pediatric patients after ECMO model [3, 5, 7]. These studies were primarily cross-sectional, mono-disciplinary, and only studied a small population of interest, like neonates, congenital heart disease, or congenital diaphragmatic hernia [3, 5]. However, to date, literature has yet to show the successful development and implementation of an effective Pediatric ECMO discharge program in non-European healthcare models. Like most other Children’s hospitals, our hospital did not have a defined evaluation and follow-up process for all our ECMO survivors. We depended on the ICU and discharging teams to identify deficits and ensure adequate follow-up, which led to many missed opportunities for our ECMO survivors.

We propose an intentional discharge protocol, STEP, where the long-term side effects of ECMO and post-discharge follow-up are explained to caregivers. The pre-discharge assessments are confirmed to be completed, ECMO-specific recommendations are sent to primary care pediatricians, and timely and complete neurodevelopmental follow-up is scheduled. We hypothesize that STEP implementation will improve compliance with the recommended follow-up based on ELSO standards and 1987 guidelines [1, 2, 15–17] to 90% or greater.

## Methods

STEP was conducted at a tertiary-care children’s hospital in the mid-south U.S.A. as a quality improvement initiative. University of Tennessee Health Science Center Institutional Review Board approved the study on February 5, 2018, with a waiver of informed consent granted before study initiation (17-05517-XP). A multi-disciplinary team, including an intensivist, developmental-behavioral pediatrician, neurologist, neuropsychologist, and audiologist, met and reviewed the at that time current guidelines and additional literature and implemented them based on institutional resources and included more intensive neurodevelopmental follow-up (Table 1). 1997 ELSO Recommendations for ECMO Follow-Up were later revised in 2021, but we kept our guidelines unchanged for the study period. It was determined that baseline neurology consultation would be done on all patients and MRI if clinical concerns by the primary team or neurologist. Still, later, this changed to a neurology consultation if the clinical team had a clinical concern and performed if concerns were raised by the primary or neurology team. Discharge lists were kept by an MRI ICU team and sent to neuropsychologists, who coordinated with developmental behavioral pediatrics to avoid duplicating tests and clinic visits. Recommendations for audiological evaluation, PT, OT, and SLP were given to the discharging team and parents. A letter with the ELSO recommendations was given to the parent and mailed to the PCP.

**Table 1:** STEP follow-up timeline.

Age at Decannulation (Years-months)	Evaluation timepoint*	Tests administered
0 to 3–11	1st Birthday	Bayley-III
	2nd Birthday	Bayley-III
	3rd Birthday	WPPSI-4; PPVT-4; EVT-2; ABAS-3; BASC-3
	5th Birthday	WPPSI-IV; PPVT-4; EVT-2; ABAS-3; BASC-3; ChAMP; VMI-VI; Purdue
4–0 to 5–11	6 months post-discharge	WPPSI-IV; PPVT-4; EVT-2; ABAS-3; BASC-3; ChAMP; VMI-VI; Purdue
	18 months post-discharge	WPPSI-IV; PPVT-4; EVT-2; ABAS-3; BASC-3; ChAMP; VMI-VI; Purdue
6–0 to 15–11	6 months post-discharge	WISC-5; PPVT-4; EVT-2; ABAS-3; BASC-3; ChAMP; VMI-VI; Purdue
	18 months post-discharge	WISC-5; PPVT-4; EVT-2; ABAS-3; BASC-3; ChAMP; VMI-VI; Purdue
16–0 to Adulthood	6 months post-discharge	WAIS-IV; PPVT-4; EVT-2; ABAS-3; BASC-3; ChAMP; VMI-VI; Purdue
	18 months post-discharge	WAIS-IV; PPVT-4; EVT-2; ABAS-3; BASC-3; ChAMP; VMI-VI; Purdue

* All patients receive further evaluations as needed.

Bayley-III Scales of Infant and Toddler Development (Bayley-III).

We Wechsler Preschool and Primary Scales of Intelligence – Fourth Edition (WPPSI-IV).

Peabody Picture Vocabulary Test-Fourth Edition (PPVT-4).

Expressive Vocabulary Test-2nd Edition (EVT-2).

Adaptive Behavior Assessment System, Third Edition (ABAS-3).

Behavior Assessment System for Children, Third Edition (BASC-3).

Child and Adolescent Memory Profile (ChAMP).

Beery Visual Motor Integration Test, Sixth Edition (VMI-VI).

Purdue Pegboard Test (Purdue).

Wechsler Intelligence Scale for Children, Fifth Edition (WISC-5).

Wechsler Adult Intelligence Scale, Fourth Edition (WAIS-IV).

The primary aim was to measure compliance with our institutional discharge protocol based on the 1997 ELSO Recommendations for ECMO Follow-Up. These include neurology and audiology evaluation, P.T., O.T., S.L.P. consultation, and neuroimaging. Secondary aims included timely evaluation for developmental delay post-ECMO through neuropsychological examination (Table 1).

All pediatric ECMO patients one day to 17 years of age who survived hospital discharge before and after program implementation between January 2011 and April 2020 were included in the study. Patients were excluded if they underwent ECMO cannulation and/or decannulation at another institution and those transferred to another institution after ECMO. The study excluded non-English-speaking patients due to inconsistent translation services for obtaining consent and program explanation. Patients with delayed follow-up (*n* = 10) during the COVID-19 pandemic due to hospital logistics and restrictions were excluded from the outpatient analysis. The database included patient demographics, basic ECMO characteristics, information about inpatient neuroimaging and neurology evaluation; audiology, speech and language pathology (S.L.P.), physical therapy (P.T.), and occupational therapy (O.T.) inpatient and outpatient referrals; and neurodevelopmental testing and referrals.

Patients were referred to a single pediatric intensivist (R.S.) upon cannulation via automatic ECMO alert. The STEP intensivist reviewed the chart, educated the family on the long-term impacts of ECMO, and coordinated care with consulting specialists and therapists as the patient neared discharge. STEP established an outpatient neurodevelopmental follow-up schedule for age groups and defined time-appropriate testing (Table 1). After family education, the STEP intensivist placed a consultation note in the electronic medical record, and the primary care physician received a letter with long-term care recommendations. Caregivers received an abbreviated version of these recommendations and/or a STEP brochure.

We estimated follow-up compliance of 55% before and an expected 90% compliance after STEP implementation. Considering an alpha of 0.05 and a power of 80%, the calculated sample size for each group of ECMO survivors pre and post-intervention was 24 patients. Demographic data and information regarding evaluations and referrals before and after STEP implementation were described using medians and interquartile ranges (IQR) for continuous variables and frequencies and percentages for categorical variables. To ascertain differences before and after STEP implementation, chi-square and Fisher exact tests were used for binomial data, and the Mann-Whitney test was used for continuous data. *p*-values were 2-sided, and *p* < 0.05 was considered statistically significant. Statistical analyses were performed using S.A.S. (version 9.4, S.A.S. Institute Inc., NC, U.S.A.).

## Results

**Figure 1:**
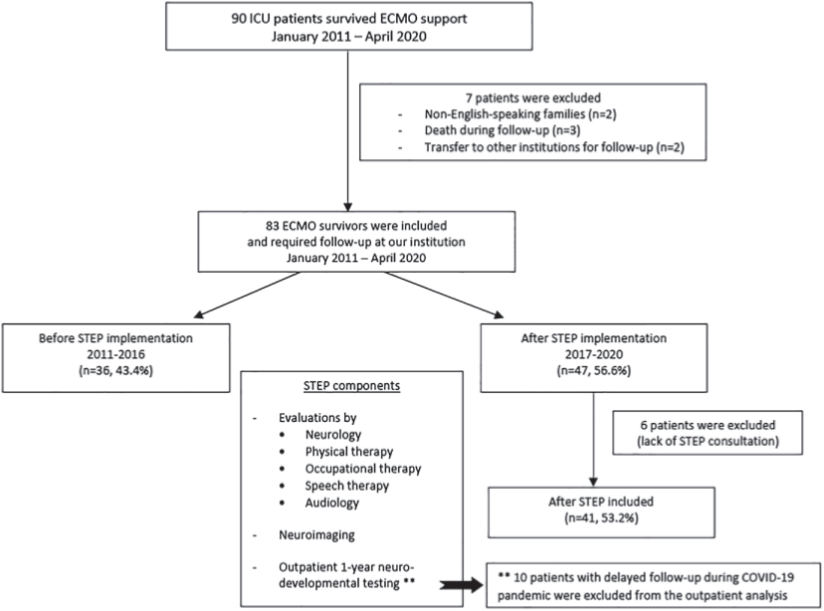
Summary of STEP follow-up.

A total of 186 neonates and children needed ECMO between January 2011 and April 2020, with 90 (48.3%) surviving ECMO decannulation. Of those, seven patients were excluded due to death between ECMO decannulation and hospital discharge (*n* = 3), due to transfer to another facility after ECMO support (*n* = 2), and non-English speaking families (*n* = 2). Of the remaining 83 ECMO survivors, 36 (43.4%) were before and 47 (56.6%) after the implementation of STEP. Six of these 47 ECMO survivors were excluded after STEP implementation due to a lack of in-hospital STEP consultation. Thus, the study included 77 ECMO survivors (36 before and 41 after STEP implementation) to hospital discharge (Fig. 1). The median age at ECMO cannulation was one month (IQR, 0.1–21.5), with 49.4% of patients being neonates and 49.4% male. Most patients were African American (58.4%), reflecting the city’s demographics [18].

Respiratory support as an indication for ECMO and veno-arterial (V.A.) ECMO cannulation modes were the most prevalent in the cohort (75.3% and 62.3%, respectively). The median ECMO duration was seven days (IQR, 3–10), and the median length of hospital stay after decannulation was 34.5 days (IQR, 23.7–71.5). No significant differences were observed in baseline characteristics between patients before and after STEP implementation, except for more non-neonatal pediatric patients (36.1% vs. 63.4%, *p* = 0.01) and more frequent veno-arterial cannulation (44.4% vs. 78%, *p* = 0.002) after STEP implementation (Table 2).

**Table 2:** Demographic characteristics.

	Total cohort (*n* = 77) 2011–2020	Before STEP (*n* = 36) 2011–2016	After STEP (*n* = 41) 2017–2020	*p*-value
Age (months)	1 (IQR, 0.1–21.5)	0.25 (IQR, 0.06–8.75)	2 (IQR, 0.11–26)	0.11
Age group				**0.01**
Neonates (<30 days)	38 (49.4%)	23 (63.9%)	15 (36.6%)	
—Pediatric (≥30 days)	39 (50.6%)	13 (36.1%)	26 (63.4%)	
Race				0.6
—Caucasian	22 (28.6%)	12 (33.3%)	10 (24.4%)	
—African American	45 (58.4%)	19 (52.7%)	26 (63.4%)	
—Other	10 (13%)	5 (14%)	5 (12.2%)	
Gender				0.2
—Male	38 (49.4%)	15 (41.7%)	23 (56%)	
—Female	39 (50.6%)	21 (58.3%)	18 (44%)	
ECMO type				**0.002**
—VA	48 (62.3%)	16 (44.4%)	32 (78%)	
—VV	29 (37.7%)	20 (55.6%)	9 (22%)	
Type of support				0.34
—Pulmonary	58 (75.3%)	25 (69.4%)	33 (80.5%)	
—Cardiac	14 (18.2%)	9 (25%)	5 (12.2%)	
—ECPR	5 (6.5%)	2 (5.6%)	3 (7.3%)	
ECMO duration (days)	7 (IQR, 3–10)	6 (IQR, 3–10)	7 (IQR, 3.5–11)	0.36
LOS since decannulation (days)	34.5 (IQR, 23.7–71.5)	30 (IQR, 21.5–60.75)	42 (IQR, 27.2–82.7)	0.13

^*^ECMO: extracorporeal membrane oxygenation, ECPR: extracardiac pulmonary resuscitation, IQR: interquartile range, LOS: length of stay, STEP: Standardizing Therapies after ECMO Program, VA: veno-arterial, VV: veno-venous.

We found a significant increase in inpatient evaluations by neurology (52.7% vs. 75.6%, *p* = 0.03) and audiology (66.7% vs. 87.8%, *p* = 0.02). Patients discharged after STEP implementation had significantly more referrals for audiology (66.6 vs. 95.1%, *p* = 0.002), P.T. (63.8% vs. 95.1%, *p* = 0.001), O.T. (63.8% vs. 95.1%, *p* = 0.001) and S.L.P. (55.5% vs. 95.1%, *p* < 0.001) (Table 3).

**Table 3:** Evaluations and referrals at discharge.

	Total cohort (*n* = 77) 2011–2020	Before STEP (*n* = 36) 2011–2016	After STEP (*n* = 41) 2017–2020	*p*-value
**Inpatient evaluations**				
Neurology	41 (53.2%)	19 (52.7%)	31 (75.6%)	**0.03**
Neuro Imaging	63 (81.8%)	31 (86.1%)	32 (78%)	0.36
Audiology	60 (77.9%)	24 (66.7%)	36 (87.8%)	**0.02**
Physical therapy	69 (89.6%)	30 (83.3%)	39 (95.1%)	0.13
Occupational therapy	66 (85.7%)	30 (83.3%)	36 (87.8%)	0.74
Speech therapy	66 (85.7%)	29 (80.5%)	37 (90%)	0.32
**Referrals placed at discharge**				
Audiology	63 (81.8%)	24 (66.6%)	39 (95.1%)	**0.002**
Physical therapy	62 (80.5%)	23 (63.8%)	39 (95.1%)	**0.001**
Occupational therapy	62 (80.5%)	23 (63.8%)	39 (95.1%)	**0.001**
Speech therapy	59 (76.6%)	20 (55.5%)	39 (95.1%)	**<0.001**

There was a significant increase in the completion of neurodevelopmental testing (38.8% vs. 74.2%, *p* < 0.001) and in time-appropriate completion (71.4% vs. 95.6%, *p* = 0.03) after STEP implementation. Neuropsychological referrals included neurodevelopmental clinics, state early intervention programs, and neonatal infant follow-up programs. We identified deficits in 24 of 37 patients (64.8%) who completed neurodevelopmental testing, with no difference before vs. after STEP implementation (64.2% vs. 65.2%) (Table 4). Deficits were grouped as global developmental delay (70.8%), attention-deficit/hyperactivity disorder (ADHD) (12.5%), language disorder (8.3%), mild intellectual disability (4.2%), and learning difficulty (4.2%).

**Table 4:** Neurodevelopmental testing.

	Total cohort (*n* = 67) 2011–2020	Before STEP (*n* = 36) 2011–2016	After STEP (*n* = 31) 2017–2020	*p*-value
Complete	37 (48%)	14 (38.8%)	23 (74.2%)	**<0.001**
Incomplete	8 (12%)	2 (5.6%)	6 (19.3%)	
Not referred	22 (32.8%)	20 (55.6%)	2 (6.5%)	
**For patients with complete neurodevelopmental testing**				
Time-appropriate	32 (86.5%)	10 (71.4%)	22 (95.6%)	**0.03**
Not time-appropriate	5 (13.5%)	4 (28.6%)	1 (4.4%)	

^*^10 patients were excluded from the analysis due to delays in obtaining hospital appointments during the beginning of the pandemic.

## Discussion

Our study is the first to describe the feasibility and effectiveness of an intentional discharge pathway for ECMO survivors in a non-European healthcare model. It provides a roadmap for single centers with limited resources to develop a comprehensive pediatric post-ECMO follow-up. The primary finding is that STEP successfully increases inpatient and outpatient compliance with STEP follow-up guidelines. Patients receiving complete and time-appropriate neurodevelopmental testing after initiating the STEP were statistically significant. It allowed for early identification of deficits and referral for appropriate therapies. We observed a substantial increase in neurology and audiology inpatient evaluations. There was a significant increase in P.T., O.T., S.L.P., and audiology referrals at discharge. The lack of increase in neuroimaging was attributed to our institution’s guidelines that limited neuroimaging use for patients with clinical concerns.

The secondary finding is that rates of developmental delay are consistent with published literature [5] and are similar in before and after STEP groups with improved timeliness of evaluation. However, current literature demonstrates that increased neurocognitive deficits appear with age, and future follow-ups could reveal more deficiencies. A response bias, in which patients with concerns are more likely to come to appointments, could be present but would be expected across both groups. Neurodevelopmental testing was decentralized before STEP implementation, but STEP provides standardized referral routes for patients to a neuropsychologist with expertise in ECMO outcomes and prompt access to resources. The evolution of ECMO indications to include children with higher severity of illness and additional pre-existing comorbidities may lead to increased deficits in ECMO survivors. Therefore, a robust ECMO follow-up program is essential to provide optimal care for ECMO survivors.

We noted a difference between the before and after groups in the number of pediatric patients consistent with the temporal evolution of indications and use in the pediatric population worldwide [19] and increased V.A. cannulations due to the withdrawal of Origen double VV cannula from the market and lack of an alternative product in the neonatal population [20]. However, these changes do not affect ECMO follow-up practices. Patients during the COVID-19 period who lacked STEP consult and non-English speaking families were excluded from the analysis to ensure we looked at the results of the STEP program. They were provided with the appropriate follow-up services and Neuropsychological follow-up.

We now realize that there are long-term effects of the ICU stay and the supportive therapies, surgeries, and treatments on the patient and their families [21–23]. Comprehensive follow-up programs with early identification of deficits and treatments have been shown to make a difference in the long term after congenital heart surgery and acquired neurological injury [24–26]. Multiple society recommendations and guidelines strongly recommend using a longitudinal and multidisciplinary follow-up [1, 15, 27, 28]. ECMO patients are at high risk of developing neurological injury, and current evidence and guidelines oblige us to provide optimal post-discharge follow-up. Our study adds to this literature and shows that optimal ECMO follow-up and neurodevelopmental assessment and treatment can be provided in the US healthcare model.

The development of STEp needed consultation and buy-in from stakeholders to ensure its long-term success. We utilized the existing relationships between ECMO, Congenital heart surgery, and Neurodevelopment programs to gain access to personnel, services, and clinic time. Our ECMO, ICU, and hospital leadership saw value in this process and supported it through multiple leadership and personnel changes and COVID disruption. Our model may be supplemented by nurses, ECMO team members, or physician extenders – advanced nurse practitioners, nurses, or physician assistants to coordinate care and family education and discussions depending on the resources available at the local institution. A successful program will need a dedicated program leader who understands the importance of a follow-up program to meet the needs of ECMO survivors.

Study limitations include the small sample size and single-center approach. Our study describes the development of post-ECMO care programs in the US health system and may be less relevant to other healthcare models. The neurodevelopmental baseline of patients before ECMO could not be assessed. Therefore, it limits our understanding of whether the neurodevelopmental changes were due to the primary etiology or ECMO. Compliance with outpatient therapy referrals could not be confirmed due to decentralized follow-up. The COVID-19 pandemic resulted in hospital, staffing, and personal protective equipment limitations, leading to delayed screening and outpatient follow-up. Several patients lacking STEP consults were excluded, highlighting the importance of a larger team to reach all patients. ECMO technology and indications have evolved over the study period, which might have led to differences in the two study populations; however, the practice and implementation of the follow-up process have remained the same.

In conclusion, our study shows that an intentional discharge protocol is feasible and successful for the ECMO population. Implementation of small and methodical steps to identify and provide early intervention for developmental deficits can improve patients’ long-term outcomes [14].

## Data Availability

The data supporting the study's findings are available upon request from the corresponding author, HSS.
